# Orchestrating movement: the role of Caveolin-1 in migration and metastasis

**DOI:** 10.1186/s12943-025-02469-6

**Published:** 2025-10-23

**Authors:** Jiri Navratil, Martina Raudenska, Monika Kratochvilova, Jan Balvan, Yoav David Shaul, Michal Masarik

**Affiliations:** 1 Department of Pathological Physiology, Faculty of Medicine, Masaryk University, Kamenice 5, Brno, CZ-625 00 Czech Republic; 2 Department of Physiology, Faculty of Medicine, Masaryk University, Kamenice 5, Brno, CZ-625 00 Czech Republic; 3 Department of Biochemistry and Molecular Biology, Faculty of Medicine, The Institute for Medical Research Israel-Canada, Hebrew University of Jerusalem, Jerusalem, Israel; 4 International Clinical Research Center, St. Anne’s University Hospital, Brno, 60200 Czech Republic; 5 Institute of Pathophysiology, First Faculty of Medicine, Charles University, U Nemocnice 5, Prague, CZ-128 53 Czech Republic

**Keywords:** Caveolin-1, Cancer, Metastasis, Modes of migration, Caveolae

## Abstract

Cancer metastasis is a complex, multi-step process that accounts for the majority of cancer-related deaths. Cell motility and directional migration are central to these processes. Cell migration’s molecular mechanisms and metastatic disease progression are strongly correlated, and Caveolin-1 (CAV1) is expected to be involved in metastasis based on its role in migrating cells. In early-stage cancers, CAV1 typically suppresses tumour growth by inhibiting cell proliferation and stabilising cellular signalling, and its downregulation or loss is commonly linked to tumour initiation. However, in advanced cancers, CAV1 expression is often upregulated and facilitates tumour progression by enhancing cell migration, invasion, metastasis, and resistance to therapy. Consequently, CAV1 has emerged as a critical mediator in transitioning from localised tumour growth to metastatic spread. However, the context-dependent roles of CAV1 make it difficult to understand its role in metastasis. This review aims to provide a comprehensive overview of the involvement of CAV1 in different modes of cancer cell migration and metastasis. We discuss its molecular functions, context-dependent roles, and interactions with key signalling pathways, including extracellular vesicle signalling, that control cell movement, shedding light on its complex contribution to cancer progression.

## Introduction

Caveolin-1 (CAV1) is a multifunctional scaffolding protein that plays a complex role in many cellular processes. As the key component of caveolae—small, flask-shaped, cholesterol-rich invaginations of the plasma membrane—CAV1 is involved in membrane organisation, signal transduction, and cellular homeostasis. However, CAV1 functions extend beyond caveolae, as it modulates diverse signalling pathways and physiological processes, making it a key player in both normal cellular function and pathological conditions [1]. CAV1 is a member of the mammalian caveolin family, encoded by the *CAV1* gene, consisting of four exons on chromosome 7q31.2. This gene gives rise to two protein isoforms: a 24-kDa α isoform comprising amino acid residues 1–178, and a shorter 21-kDa β isoform containing amino acid residues 32–178 [2]. The β isoform is produced through alternative translation initiation from the full-length mRNA [2] or from a shorter transcript variant of mRNA (5′V mRNA) [3]. Some studies suggest the existence of two distinct caveolae populations, with a higher α/β isoform ratio observed in deep caveolae compared to shallow ones. Notably, the α isoform seems more effective in promoting caveolae formation [4]indicating that the two may have distinct physiological functions. Furthermore, the phosphorylation site on tyrosine 14 (Y14), critical for several CAV1 functions, is exclusive to the α isoform [5]. Therefore, this review will primarily focus on the α isoform.

CAV1 is a membrane-sculpting protein that oligomerises to generate caveolae. Phosphorylation of Y14 in CAV1 induces conformational changes that spatially separate individual CAV1 molecules within the oligomer. This structural rearrangement is believed to alter the conformation and accessibility of the caveolin scaffolding domain, thereby facilitating its interaction with other proteins [6]. Y14 phosphorylation also promotes *CAV1* and *CAVIN1* expression by triggering actin-dependent mechanotransduction and reducing the binding affinity of the transcription factor Egr1 (early growth response-1) to the promoters of *CAV1* and *CAVIN1* genes. Since Egr1 binding represses the transcription of these genes, its dissociation leads to transcriptional derepression and upregulation of both *CAV1* and *CAVIN1*[7]. In the absence of CAVIN1 (also known as PTRF), CAV1 cannot form caveolae and instead assembles into non-caveolar membrane microdomains referred to as scaffolds. These CAV1 scaffolds participate in focal adhesions, growth factor receptor signalling, and the regulation of raft-dependent endocytosis [8].

Both mechanical cues and biochemical signalling influence the dynamic behaviour of caveolae (flattening/internalisation), and these pathways are often interdependent. Mechanosensation, such as increased membrane tension due to external forces or osmotic stress, drives caveolae flattening or internalisation [9, 10]. Phosphorylation of CAV1 at tyrosine 14 is a key regulatory event that influences the structural integrity and signalling functions of caveolae. Under mechanical stress, high glucose, or growth factor stimulation, CAV1 is phosphorylated, which weakens the oligomeric interactions that stabilise caveolae at the plasma membrane [6, 11]. This leads to caveolae flattening or internalisation, particularly at dynamic sites like the leading edge of migrating cells [12]. Cortical tension, generated by the actin cytoskeleton beneath the plasma membrane, plays a crucial role in regulating caveolae internalisation. CAV1 was shown to bind actin cross-linking proteins, such as filamin A, which regulates caveolae internalisation and trafficking [13].

The role of CAV1 in cancer is highly context-dependent. In the early stages of cancer development, CAV1 functions as a tumour suppressor by inhibiting cell proliferation, promoting apoptosis, and stabilising cellular signalling. Its downregulation or loss is frequently associated with tumour initiation and poor clinical prognosis. In contrast, in advanced cancers, CAV1 expression is often upregulated and contributes to tumour progression by enhancing cell migration, invasion, metastasis, and resistance to therapy [14, 15]. This functional shift is thought to be caused by changes in signalling dynamics within the tumour microenvironment [1] and the molecular landscape of tumour cells. For example, the effect of CAV1 signalling may be significantly affected by E-cadherin expression [14–16]. Díaz-Valdivia et al. revealed that CAV1 phosphorylation of Y14 is diminished in cells expressing E-cadherin by non-receptor tyrosine phosphatase type 14 (PTPN14) that is active in CAV1/E-cadherin complexes. PTPN14 overexpression suppresses CAV1-induced migration, invasion, and metastasis of cancer cells [14]. Increased CAV1 expression in the absence of E-cadherin was found to work as a “molecular switch” that promotes metastasis [16]. Loss of E-cadherin expression is a hallmark of the epithelial-to-mesenchymal transition (EMT), a key program in cancer metastasis, fibrosis, or wound healing. EMT involves the loss of epithelial characteristics, such as cell-cell adhesion, and the acquisition of mesenchymal traits, including increased motility and invasiveness. During EMT, E-cadherin is replaced by N-cadherin, which leads to the disruption of adherent junctions and promotes cancer cell dissemination [17]. Importantly, CAV1 and N-cadherin are upregulated in metastatic melanoma cell lines [16]. CAV1 expression is frequently induced during EMT and has been shown to impact cancer cell adhesion [18].

Cancer metastasis is a highly complex, multi-step process that is the leading cause of cancer-related mortality worldwide. It involves a coordinated cascade of events, including the detachment of tumour cells from the primary site, migration through the extracellular matrix, invasion of surrounding tissues, intravasation, survival in circulation, extravasation, and subsequent colonisation of distant organs. Cell motility and directional migration are at the heart of these processes. Cell migration modes are broadly classified into single-cell migration modes, including mesenchymal, amoeboid, and osmotic engine types, and collective migration modes, which include coordinated movement of cell strands, sheets, or clusters [19]. Although significant advances have been made in elucidating the molecular mechanisms of cancer cell migration, much remains to be understood. In the context of cancer metastasis, CAV1 has emerged as a key player, particularly in mediating the transition from localised tumour growth to widespread dissemination. However, the functional role of CAV1 is highly context-dependent, influenced by the cellular and molecular environment, thereby complicating efforts to define its contribution to metastatic progression.

Although cancer cell migration and CAV1 have both been extensively studied in the context of metastasis and tumour progression, the intersection between the two, specifically, how CAV1 orchestrates migratory behaviour during metastatic dissemination, remains insufficiently explored. This review aims to address this critical knowledge gap by providing a comprehensive synthesis of the current understanding of the mechanistic role of CAV1 in regulating various modes of cancer cell migration, as well as CAV1’s functional contribution to metastatic progression. We emphasise the context-dependent functions of CAV1 and detail its involvement in processes such as cytoskeletal remodelling, membrane tension regulation, and the coordination of membrane protrusions that are essential for cell motility. These include lamellipodia, filopodia, lobopodia, podosomes, invadopodia, and pseudopodia. Furthermore, we examine CAV1’s role in modulating the tumour microenvironment, particularly within the pro-metastatic stroma, including extracellular vesicle-mediated signalling. Integrating these insights, this review underscores the pivotal role of CAV1 in linking cell motility to metastatic competence across cancer types.

## CAV1 in mesenchymal migration

Mesenchymal migration represents a highly polarised, adhesion-dependent mode of cell motility characterised by elongated morphology, integrin-mediated interactions with the extracellular matrix (ECM), and coordinated proteolytic remodelling of the surrounding stroma. Mesenchymal migration is frequently observed in tumours derived from connective tissues or the bone marrow, as well as in certain poorly differentiated epithelial cancers. This migratory strategy is commonly observed during the initial phases of invasion, particularly in epithelial-derived tumours undergoing EMT. Through mesenchymal migration, cancer cells acquire the ability to navigate tissue barriers, degrade basement membranes, and disseminate locally and systemically. This mode of movement is closely linked to early metastatic events, including stromal infiltration and intravasation [20]. In dense or spatially confined ECM environments, cells rely on matrix metalloproteinases (MMPs) and serine proteases to degrade ECM components and enable forward movement. A key structural feature of mesenchymal migration is the anterior positioning of the microtubule-organising centre (MTOC) relative to the nucleus, which facilitates the targeted delivery of protease-containing vesicles to the leading edge of the cell. This spatial organisation is critical for efficient ECM degradation [21]. The nucleus, as the stiffest cellular component, plays a rate-limiting role in migration. In collagen-rich matrices, tumour cells become immobilised when pore sizes fall below approximately 7μm². Under such conditions, cell migration is only possible through MMP-dependent ECM remodelling [22]. CAV1 contributes to this process by interacting with CD147, a known regulator of MMP activity. This interaction reduces CD147 clustering and attenuates its ability to induce MMP-1 activity. Notably, the formation of the CD147-CAV1 complex is cholesterol-dependent, implicating the involvement of lipid rafts or caveolae in its assembly [23]. CAVIN1 and CAV1 are essential components for forming the core structure of caveolae. While metastatic PC3 prostate cancer cells express high levels of CAV1, they exhibit low expression of CAVIN1. Ectopic expression of CAVIN1 decreased MMP9 production in PC3 cells [24]. Although CAVIN1 expression is low in PC3 cells, it is sufficient for the formation of some caveolae [25]. However, other signalling functions managed by CAVIN1 may be disturbed, such as inhibition of MMP-9 production and ECM degradation [24].

Invadopodia and podosomes are specialised, proteolytically active membrane protrusions that play a critical role in ECM degradation and are closely associated with invasive cellular behaviour. Invadopodia are particularly prominent in cancer cells and facilitate invasive migration by concentrating actin filaments and proteolytic enzymes. CAV1 is a key regulator of invadopodia formation due to modulating membrane cholesterol levels [26]. Functional studies have demonstrated that CAV1 knockdown impairs invadopodia formation and ECM degradation. This defect can be rescued by the expression of a non-phosphorylatable CAV1 mutant (Y14F), whereas a phosphomimetic mutant (Y14D) fails to restore invadopodia function. These findings indicate that while CAV1 is essential for invadopodia formation, phosphorylation at Y14 negatively regulates this process. Mechanistically, phosphorylation of CAV1 at Y14 creates a docking site for the SH2 domain of C-terminal Src kinase (Csk), which subsequently inactivates Src. As the Src activation is crucial for invadopodia formation and activity, this phosphorylation-dependent inhibition serves as a regulatory checkpoint [26]. Furthermore, CAV1 responds to mechanical cues such as shear stress. Under these conditions, CAV1 clustering within lipid rafts triggers the PI3K/Akt/mTOR signalling pathway, further enhancing invadopodia formation and promoting metastasis potential in breast carcinoma cells [27].

Podosomes are dynamic, actin-rich structures that contribute significantly to cell adhesion, migration, and ECM degradation. They are predominantly found in highly motile cell types, including macrophages, osteoclasts, v-Src transformed fibroblasts, and cancer cells. Podosomes host structural and signalling molecules like F-actin, Src, PI3-kinase, integrins, and MMPs (e.g. membrane-anchored collagenase MT1-MMP). This molecular architecture enables them to mediate focal ECM degradation efficiently [28]. CAV1 is implicated in the regulation of podosome formation through its control of key molecules such as Src and Cdc42, as well as integrin-mediated adhesion, critical for the assembly and functionality of podosomes [29, 30].

**Fig. 1:**
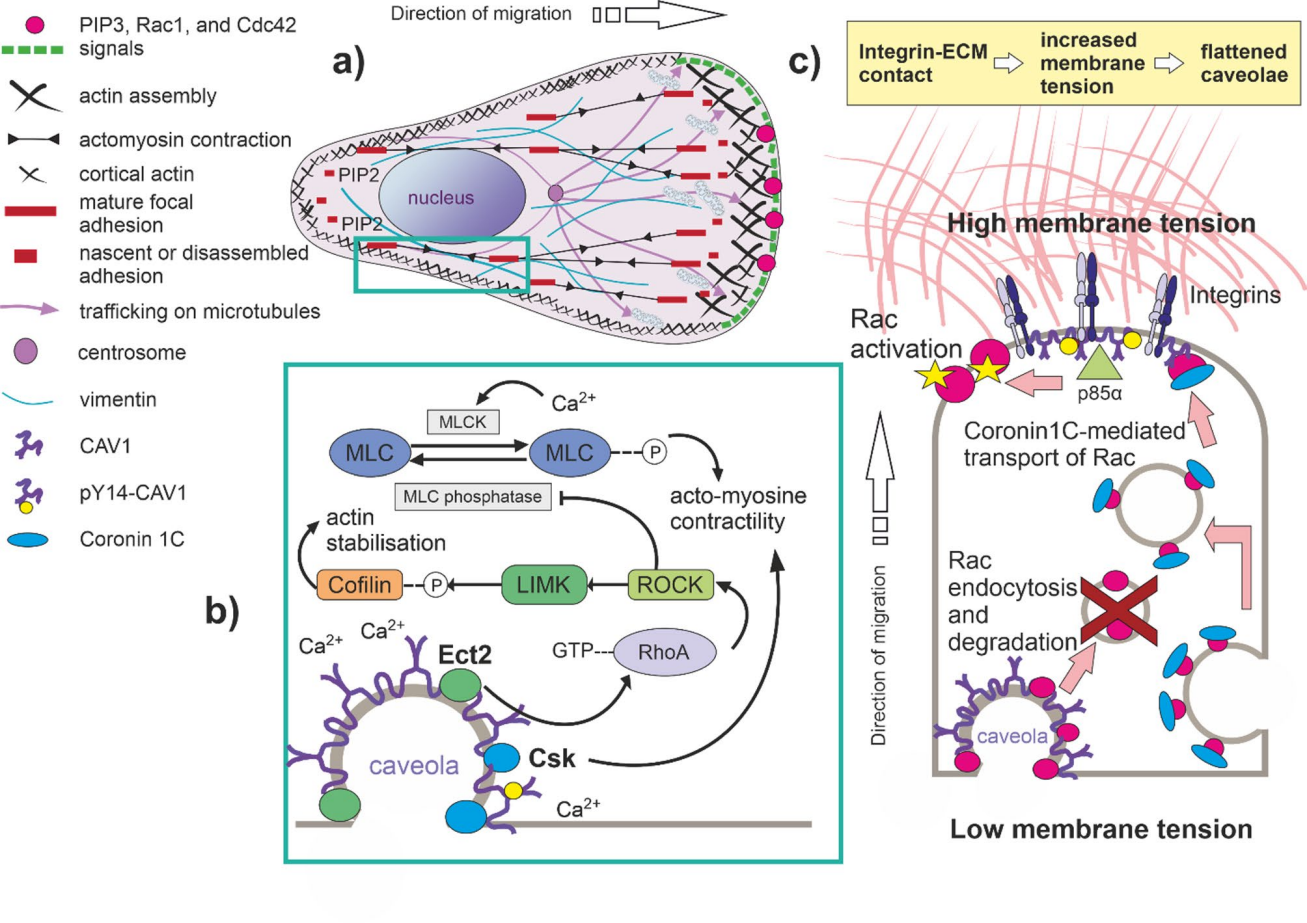
Lamellipodia-based mesenchymal migration. **a** In lamellipodia-based mesenchymal migration, the microtubule organising centre and mitochondria polarise toward the front to support degradation of ECM and energy-demanding processes. Polarised activity of Rac1 and Cdc42, and PIP3 gradients define the leading edge, promoting lamellipodia formation and focal adhesion assembly. PI3K activity at the front and PTEN at the rear maintain this polarity, while actomyosin contractility retracts the trailing edge. **b** During migration, caveolae flatten or relocate to the cell rear (turquoise rectangle in a) and b)), where they promote ATP-stimulated calcium release from the ER. This triggers RhoA and GEF-H1 oscillations that regulate microtubule dynamics and cell speed [51]. Calcium also activates MLCK, leading to myosin II-driven contraction [52]. Caveolae recruit Ect2, activating the RhoA-ROCK1/PKN2 pathway and modulating actin organisation. Y14-phosphorylated CAV1 recruits Csk, inhibiting Src and influencing cytoskeletal remodelling and myosin light chain (MLC) phosphorylation. **c** Lamellipodia increase membrane tension, triggering caveolae flattening or rearward translocation. At the rear, caveolae aid Rac1 degradation, while at the front, pY14-CAV1 promotes Rac1 activation by recruiting p85α and sustaining Rab5 activity. Additionally, Coro1C helps redistribute Rac1 to the leading edge for reactivation. ECM = extracellular matrix; PIP3 = phosphatidylinositol 3,4,5-trisphosphate [PI(3,4,5)P3]; PIP2 = phosphatidylinositol 4,5-bisphosphate [PI(4,5)P2]; PTEN = Phosphatase and tensin homolog; PI3Ks = Phosphatidylinositol-3-kinases; pY14-CAV1 = CAV1 phosphorylated on tyrosine 14; Ect2 = RhoA guanine nucleotide exchange factor; Csk = C-terminal Src kinase

Upon stimulation, the region with the highest PI3K, Cdc42, Par6, and Rac1 activity becomes the leading edge of the migrating cells. This front-rear polarity is characterised by elevated levels of phosphatidylinositol 3,4,5-trisphosphate (PIP3) and the assembly of focal adhesions at the cell front (see Fig. 1). PIP3 levels are regulated by the opposing actions of phosphatidylinositol 3-kinase (PI3K) which generates PIP3 at the leading edge, and the phosphatase PTEN, which degrades PIP3 to PIP2 at the trailing edge [31–33]. This spatially controlled PIP3 gradient and regulation of PTEN through a feedback loop are essential for actin polymerisation at the front of the cell and play a central role in gradient sensing and directional migration [34]. Nevertheless, PTEN is frequently deleted or inactivated in cancer cells. In PTEN-negative cells, a significant increase in the Rac1 and Cdc42 activity is observed [35]. Since PTEN-negative cells can migrate and maintain their directionality, this suggests the presence of compensatory mechanisms for PIP3 dephosphorylation that maintain directional migration even after PTEN loss. In this regard, PIP3 can also be dephosphorylated by PI5-phosphatases SHIP1/2 and OCRL [36]. Indeed, in murine neutrophils, PTEN deletion does not impair chemotaxis or cell polarisation in the presence of active phosphatase SHIP1[37]. Given the antagonistic relationship between Rac1 and RhoA [38]CAV1 may also contribute to restoring balance between these signalling pathways. CAV1 has been shown to positively regulate RhoA activity [39] while negatively regulating Rac1[40, 41], thereby contributing to the coordination of cytoskeletal dynamics. Additionally, PTEN loss amplifies CAV1-driven dissociation of β-catenin from membranous E-cadherin, potentially facilitating senescence bypass and metastatic process [42].

**Fig. 2:**
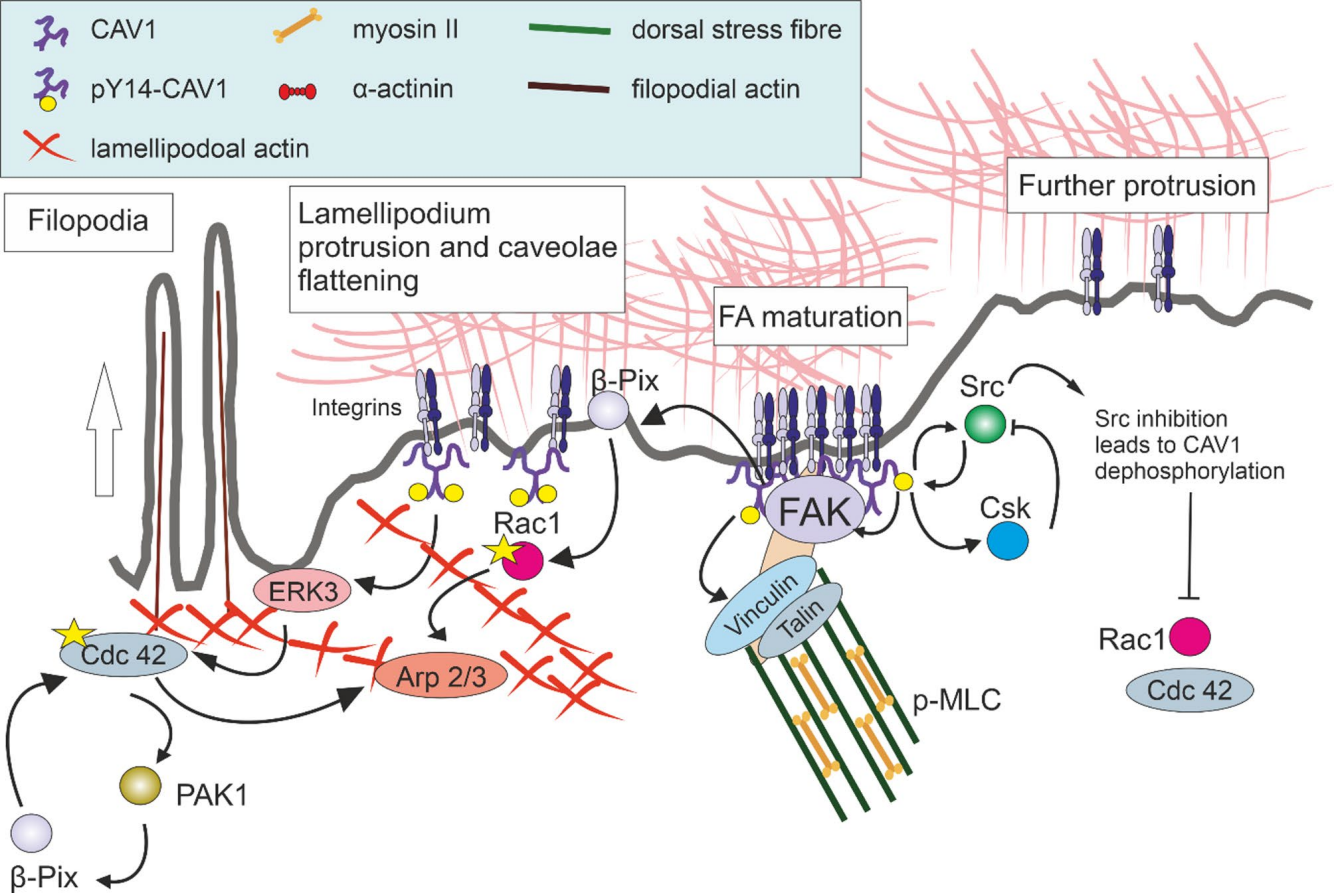
Model of CAV1 effect on focal adhesions (FAs). At the leading edge, Cdc42 drives filopodia and Rac1 promotes lamellipodia formation by activating ARP2/3, establishing polarity and actin dynamics. ERK3, acting as a GEF for Cdc42 and phosphorylating ARP3, is kept inactive by caveolar CAV1 but becomes activated upon caveolae flattening during migration. Phosphorylated CAV1 (pY14-CAV1) supports focal adhesion (FA) formation and FAK activation. Integrin signalling recruits β-Pix to FAs, where FAK phosphorylates β-Pix, further activating Rac1 and Cdc42 and inhibiting myosin II. CAV1 can inhibit Cdc42, but this is relieved by glucose or CAV1 phosphorylation at Y14, promoting Cdc42 activation. Activated Cdc42 stimulates PAK1, which further enhances Rac1 activation via β-Pix. pY14-CAV1 increases vinculin tension, reinforcing FAs, while also promoting Src inactivation via Csk and consequent Rac1 and Cdc42 deactivation. Csk operates as a “gatekeeper” to control Src activity and prevent aberrant signalling. Following Src inhibition, FA-associated tyrosine phosphatase dephosphorylates CAV1, which stimulates the transport of CAV1 to the cell’s rear

Symmetry breaking in the cells migrating by mesenchymal movement is based on actin polymerisation at the leading edge, which drives the protrusion of the plasma membrane to form lamellipodia and filopodia (Fig. 2). Filopodia and lamellipodia sense substrate rigidity and regulate nascent adhesion formation. Filopodia (driven by Cdc42) and formins act as mechanosensors via myosin II, probing substrate stiffness and retracting on softer surfaces. Lamellipodia (formed by ARP2/3-mediated branched actin) sense rigidity through cell spreading and adhesion regulation independent of myosin II. Both structures rely on actin polymerisation to push the membrane forward, while myosin II generates retrograde actin flow, balancing forces for mechanosensation and adhesion dynamics [43]. The CAV1 Y14 phospho-deficient mutation or CAV1 knockout triggered significant AMPK phosphorylation, reducing RhoA-myosin II activity and increasing Rac1-PAK1-Cofilin activity. This caused disordered stress fibres and prominent lamellipodia formation [44]. Cdc42 and Rac1 regulate cell polarity and coordinate actin polymerisation at the leading edge by activating ARP2/3 (the actin nucleator complex). ERK3, an atypical member of the mitogen-activated protein kinases (MAPKs), directly interacts with Rac1 and Cdc42 and is essential for their activation. ERK3 functions as a nucleotide exchange factor for Cdc42 and phosphorylates the ARP3 subunit of the ARP2/3 complex at S418, facilitating actin polymerisation. Accordingly, ERK3 depletion inhibited basal and EGF-induced activation of Rac1 and Cdc42, disrupted F-actin maintenance, suppressed filopodia formation, and impaired epithelial cell migration [45]. CAV1 within caveolae may maintain ERK in an inactive state. However, upon translocation to non-caveolar sites in response to stretch stress, CAV1 facilitates stretch-induced ERK activation by interacting with β1-integrins, Fyn, and Shc [46]. Overexpression of CAV1 accelerated the metastatic progression of embryonal rhabdomyosarcoma through an ERK-dependent mechanism while promoting stress fibre formation and reorganisation of the actin cytoskeleton [47]. The expression and phosphorylation levels of the CAV1/Src/ERK signalling pathway are modulated by FGFR activation and cell density [48].

Lamellipodia and filopodia formations cause a local increase in membrane tension due to the stretching of the lipid bilayer. Consequently, the cell’s leading edge exhibits dynamic changes in membrane tension that further coordinate cytoskeletal remodelling, adhesion, and intracellular signalling to facilitate directional movement [49] (see Fig. 1). The higher membrane tension at the front and the lower membrane tension at the back of migrating cells causes caveolae to flatten and be excluded from the leading edge and relocated to the back of the cell [49]. Src-dependent phosphorylation of CAV1 on Y14 promotes swelling and release of caveolae [12]. As the translocated caveolae contain components of the signalling machinery required for ATP-stimulated calcium release from the ER, they are thought to act as carriers, transporting this machinery to specific cellular locations [50]. Calcium release can induce oscillations in GEF-H1 and RhoA activity aligned with microtubule dynamics, creating a molecular clock that governs cell speed [51]. A calcium pulse can also activate myosin light chain kinase (MLCK), triggering myosin II-driven contraction of actin filaments [52]. Moreover, caveolae play a critical role in regulating Rac1 activity by mediating its endocytosis and degradation, particularly at the caveolae-rich membrane domains located at the rear of migrating cells [53]. In contrast, at the leading edge, pY14-CAV1 can promote the activation of Rab5 by facilitating its GTP loading [14]. Specifically, CAV1 sequesters p85α, a regulatory subunit of PI3K that also functions as a Rab5 GTPase-activating protein (Rab5-GAP). By binding to and inactivating p85α, CAV1 prevents Rab5 deactivation, ensuring its persistent GTP-bound state. Active Rab5 regulates early endosome dynamics and facilitates the recruitment of effector proteins, including TIAM1, a guanine nucleotide exchange factor (GEF) for Rac1. Rab5-dependent recruitment of TIAM1 to early endosomes positions it near Rac1, where it catalyses GDP for GTP exchange, activating Rac1. This localised activation of Rac1 at early endosomes initiates downstream signalling cascades that modulate the actin cytoskeleton into the actin network contained in lamellipodia [54]. Consequently, CAV1 appears to participate in both activation (by preventing Rab5 deactivation [54] and targeted Src localisation [55]) and deactivation of Rac1 (by Csk-mediated Src inactivation [56] and Rac1 degradation [40]). Csk terminates the positive feedback loop that can be formed between Src and CAV1[12, 55], thereby tightly regulating Rac1 activity during cell migration. This duality enables CAV1 to coordinate the spatial and temporal control of Rac1 signalling. However, CAV1’s role in Rac1 deactivation is likely to be more significant than its role in activation, as CAV1-deficient cells exhibit disrupted cell polarity, impaired wound healing, reduced RhoA, and increased Rac and Cdc42 activities. As a result, these cells lose directional migration and fail to respond effectively to external directional cues [49, 57]. The accelerated Rac1 activation and loss of directional movement in cells lacking CAV1 is probably due to the widespread activation of Rac1 compared to its localised activation in CAV1-expressing cells [53]. Moreover, a hyperactive mutant of Rac1 (P29S) drives lamellipodia formation but negatively regulates invadopodia formation and function [58]. Nevertheless, the interplay between CAV1, Cdc42, and Rac1 can vary depending on the cell type and the specific cellular context. For example, restoration of the wild-type phenotype in CAV1-deficient cells is possible by Src inactivation or p190RhoGAP knockdown [30, 44].

During three-dimensional (3D) migration, CAV1 predominantly localises in a cytoplasmic form at the cell front. During planar movement, CAV1 remains concentrated at the cell rear within caveolae. The phosphorylation of Y14 in CAV1 is essential for its polarisation during 3D migration [59]. Notably, unphosphorylated and phosphorylated CAV1 tend to localise at opposite poles of the cell. Phosphorylated pY14-CAV1 was co-localised with the focal adhesion marker phosphopaxillin at the leading edge of migrating cells. Conversely, a notable fraction of CAV1 with unphosphorylated Y14 was observed at the rear of migrating cells [57]. CAV1 phosphorylation at Y14 is frequently triggered by mechanical stress, such as shear stress or high ECM stiffness, and is associated with the flattening of caveolae and CAVIN1 release [60]. Caveolae formed at the rear of the cell in response to low membrane tension recruit the RhoA guanine nucleotide exchange factor Ect2, which activates the RhoA-ROCK1/PKN2 signalling pathway that regulates local F-actin organisation and contractility in this specific subcellular region, thereby facilitating rearward retraction of the cell rear [61].

### CAV1 as a regulator of focal adhesions and actomyosin contractility in mesenchymal migration

Focal adhesions (FAs) serve as mechanosensitive hubs linking the actin cytoskeleton to the ECM through integrins. These complexes initiate at the leading edge as nascent adhesions and mature under mechanical tension from actomyosin stress fibres. FA turnover is essential for motility and is tightly regulated by intracellular signalling and autophagy [62, 63]which facilitates adhesion disassembly at the trailing edge [64]. Activation of PI3Ks at the leading edge can suppress autophagy locally, thereby favouring FA formation, while autophagy remains active at the trailing edge to enable FAs disassembly [65]. Notably, cells deficient in autophagy exhibit decreased migratory activity, reduced FA disassembly, and increased FA lifespan. The autophagy receptor NBR1 has been shown to interact with FAs and recruit autophagosomes to these sites [66]. Accordingly, inhibition of autophagy reduced cell migration and invasion in thyroid cancer or lung adenocarcinoma lines [67, 68]. Finally, under oxidative stress, CAV1 phosphorylated at Y14 interacts with the BECN1/VPS34 complex via its scaffolding domain, promoting autophagosome formation [69].

More than 150 proteins have now been identified as components of FAs, reflecting their complex structural and signalling roles. These include integral membrane proteins such as integrins, syndecans, proteins linked to actin (vinculin, talin, α-actinin, filamin A, zyxin), or signalling and adaptor proteins such as Src tyrosine kinase, focal adhesion kinase (FAK), paxillin, and the integrin-linked kinase (ILK). FAs also contain transiently associated proteins modulating cell migration (p21-activated kinase (PAK), the Rho family of GTPases (Rac1, Rho), calcium-dependent protease calpain 2, and the tyrosine phosphatase SHP-2) [70].

CAV1 plays a central role in modulating FA dynamics. Phosphorylation at Y14 is necessary for the stable localisation of CAV1 at FAs. Conversely, CAV1 dephosphorylation appears to be a critical step for FA turnover, facilitating adhesion turnover and promoting cell motility [40, 71]. Phosphorylation of CAV1 at tyrosine 14 (pY14-CAV1), often Src-dependent [72]enhances vinculin tension, stabilises adhesions, and promotes forward migration [6]. Within distinct membrane domains, Src phosphorylates CAV1 at Y14, enabling the SH2 domain of activated Src to bind pY14. This interaction enhances Src’s association with the plasma membrane, preferentially directing activated Src to FAs [55]. pY14-CAV1 also interacts with FAK and regulates its activity and localisation [7] in a galectin-3/Mgat5-dependent manner. In the absence of the Mgat5/galectin lattice, pY14-CAV1 alone is insufficient to drive FAK stabilisation [73]. Upon FAK integration into FAs, its autoinhibition conformation is relieved, enabling efficient autophosphorylation and interaction with Src kinase. Src kinase subsequently phosphorylates multiple tyrosine residues in FAK, resulting in complete activation of FAK [64]. On the other hand, FAK may function as a scaffolding protein to recruit Src. In this constitution, Src can indirectly associate endophilin A2 with FAK. In this complex, Src phosphorylates endophilin A2, thereby inhibiting endocytosis of membrane-anchored collagenase MT1-MMP and enhancing ECM degradation [28] and invasive activity [74]. CAVIN1 expression in PC3 cells decreases FAK stabilisation within FAs and reduces cell motility, independent of pY14-CAV1 levels. The addition of exogenous Galectin-3 restored FAK stabilisation in FAs of CAVIN1-expressing cells and dose-dependently increased their motility to levels observed in PC3 cells lacking CAVIN1[6, 75].

Crosstalk between FAK and pY14-CAV1 enables cells to adapt to mechanical cues, fine-tuning mechanosignalling and cellular outcomes [76]. For example, mechanosensitive activation of CAV1 facilitates CAV1 clustering in lipid rafts and triggers the PI3K/Akt/mTOR signalling pathway and promotes CAV1/MT1-MMP co-localisation in invadopodia, enhancing breast cancer motility, invadopodia formation, and metastasis [27]. Laminar shear stress enhances cell motility in a CAV1-dependent manner by altering the expression of actin-associated proteins, including ROCK, p-MLC, cofilin, and filamin A. Filamin A directly binds CAV1[77] and actin, contributing to the mechanosensitivity of FAs [78]. Moreover, CAV1 overexpression resulted in filamin A mRNA and protein levels upregulation and Akt-dependent filamin A phosphorylation on Ser-2152, enhancing IGF-I-induced migration of MCF-7 cells [79].

FAs forming and maturation are crucially dependent on spatiotemporal control of intracellular signalling events (Fig. 2). In polarised cells, integrin-mediated adhesion promotes the recruitment of the GEF β-Pix to FAs at the leading edge, where FAK phosphorylates β-Pix [80]. β-Pix subsequently mediates the recruitment and localised activation of Rac1 And Cdc 42 at these sites [81, 82]. CAV1 can maintain Cdc42 in an inactive state [83]. Glucose stimulates dissociation of CAV1 from Cdc42 and promotes Cdc42-βPix binding and Cdc42 activation [82]. CAV1’s interaction with Cdc42 and Rac1 can be influenced by phosphorylation. For example, phosphorylation of CAV1 on tyrosine 14 can disrupt its interaction with Cdc42, favouring Cdc42 activation [84]. Cdc42 can bind to and activate PAK1[85], which in turn can interact with α- and β-PIX [86, 87]. These GEFs can then activate Rac1[81, 82]. FA assembly is highly responsive to matrix stiffness and is mediated by integrin activation. On softer matrices, β1 integrin and CAV1 levels decrease, with β1 integrin selectively undergoing endocytosis followed by lysosomal degradation. Disruption of lipid rafts using methyl-β-cyclodextrin, nystatin, or CAV1 knockdown via siRNA decreased cell spreading, FA assembly, and β1 integrin protein levels in cells cultured on stiff matrices. Overexpression of CAV1, particularly the phospho-mimetic mutant CAV1-Y14D, prevented the reduction in β1 integrin protein levels, cell spreading, and FA assembly induced by soft matrices. Interestingly, overexpression of an auto-clustering β1 integrin also counteracted soft matrix-induced decreases in CAV1 levels, indicating a reciprocal regulatory relationship between β1 integrin and CAV1[88]. In the absence of integrin binding to fibronectin, Rac1 is retracted from the plasma membrane by caveolae-mediated endocytosis or redistributed from the lateral membrane by Coro1C for reactivation at the leading edge [53]. Notably, pY14-CAV1 also recruits C-terminal Src kinase (Csk), which phosphorylates Src at the conserved tyrosine residue Y527 found in the C-terminal regulatory domain. This phosphorylation promotes the binding of Src to its own SH2 domain, leading to an inactive, autoinhibited conformation [89]. Csk operates as a “gatekeeper” to control Src activity and prevent aberrant signalling. The pY14-CAV1-Csk pathway can mediate cytoskeletal rearrangements and regulate myosin light chain phosphorylation in response to shear stress [90].

Strengthening and maturation of FAs need the assembly of stress fibres and Rho/ROCK-mediated contractility. Deficient phosphorylation and silencing of CAV1 significantly compromise the formation of stress fibres [91] by deactivating RhoA-dependent myosin light chain (MLC) phosphorylation [44]. Rho-dependent stress fibre formation was also blocked in Csk-deficient mouse embryonic fibroblasts, as this process required the catalytic activity of Csk [92]. Fyn kinase resident in caveolae is activated by insulin or stress and stimulates stress fibre formation [93]probably through cortical actin ring depolymerisation and the reorganisation of the cortical actin into stress fibres [94, 95]. Stress fibres, composed of actin and myosin II, generate traction forces critical for cell translocation. Actin polymerisation at the leading edge, regulated by Rac1 and Cdc42, drives protrusion formation, while RhoA/ROCK-mediated contraction retracts the rear [96]; see Fig. 1b.

In conclusion, CAV1 is crucial in maintaining the RhoA–Rac1 signalling balance, modulating cytoskeletal dynamics. RhoA activated by local calcium signalling [51, 97] enhances actomyosin contractility and rear retraction by inhibiting MLC phosphatase and promoting MLC phosphorylation [98, 99]. Loss of CAV1 impairs RhoA activation, reduces myosin light chain (MLC) phosphorylation, and disrupts contractility [49]. Caveolae contribute to this process by harbouring components essential for ATP-stimulated calcium release from the ER [50].

Aberrant CAV1 expression or activation is frequently linked to increased migration, metastasis, and associated with poor prognosis in various cancers, including oesophageal, pancreatic, prostate and hepatocellular carcinoma [100–102]. In many aggressive cancers, CAV1 enhances activation of pro-EMT pathways, increasing mesenchymal migration and invasiveness [103, 104]. However, this effect of CAV1 is context-dependent. In cancer cells with naturally low CAV1 expression, such as a pancreatic cancer cell Line Panc 10.05, CAV1 induces an epithelial phenotype and promotes cell-cell contact, with increased expression of E-cadherin and beta-catenin [105]. Furthermore, as CAV1 modulates the spatial dynamics and activation of MMPs, it may influence the sensitivity or resistance of cancer cells to MMP-targeted therapies. Given the context-dependent functions of CAV1 in cancer, a dual therapeutic strategy tailored to its expression pattern may offer clinical benefit. In early-stage tumours or certain subtypes where CAV1 is downregulated, restoring its expression may suppress proliferation, maintain epithelial architecture, and inhibit EMT and mesenchymal migration. Conversely, in advanced cancers where CAV1 is upregulated and associated with increased migration, invasion, and metastasis, targeted inhibition of CAV1 or its active forms (e.g., phosphorylated CAV1) could impair metastatic spread and sensitise tumours to therapy. For example, CAV1 knockout in 4T1 Mammary cancer cells reduced extracellular vesicle release, motility, and MMP secretion and significantly suppressed lung metastasis. Gene expression profiling revealed altered expression of 21 migration-related genes in CAV1-deficient tumours [106].

## CAV1 in amoeboid migration

Amoeboid migration plays a crucial role in metastasis due to its speed, adaptability, and independence from proteolytic remodelling of the ECM. Unlike mesenchymal migration, which requires degradation of ECM, amoeboid movement allows cancer cells to squeeze through pre-existing gaps using actomyosin-driven contractility. This makes amoeboid migration particularly advantageous in tumour microenvironments where matrix degradation is inefficient or suppressed. Generally, in soft, deformable ECM, tumour cells tend to adopt an amoeboid phenotype, while stiffer matrices promote invadopodia formation and mesenchymal migration [107]. Moreover, cancer cells can switch between mesenchymal and amoeboid modes (termed mesenchymal–amoeboid transition; MAT), enhancing their ability to invade diverse tissue environments. This plasticity contributes to therapeutic resistance, especially to MMP inhibitors, and supports rapid dissemination, making amoeboid migration a key target for anti-metastatic strategies [19, 108].

**Fig. 3:**
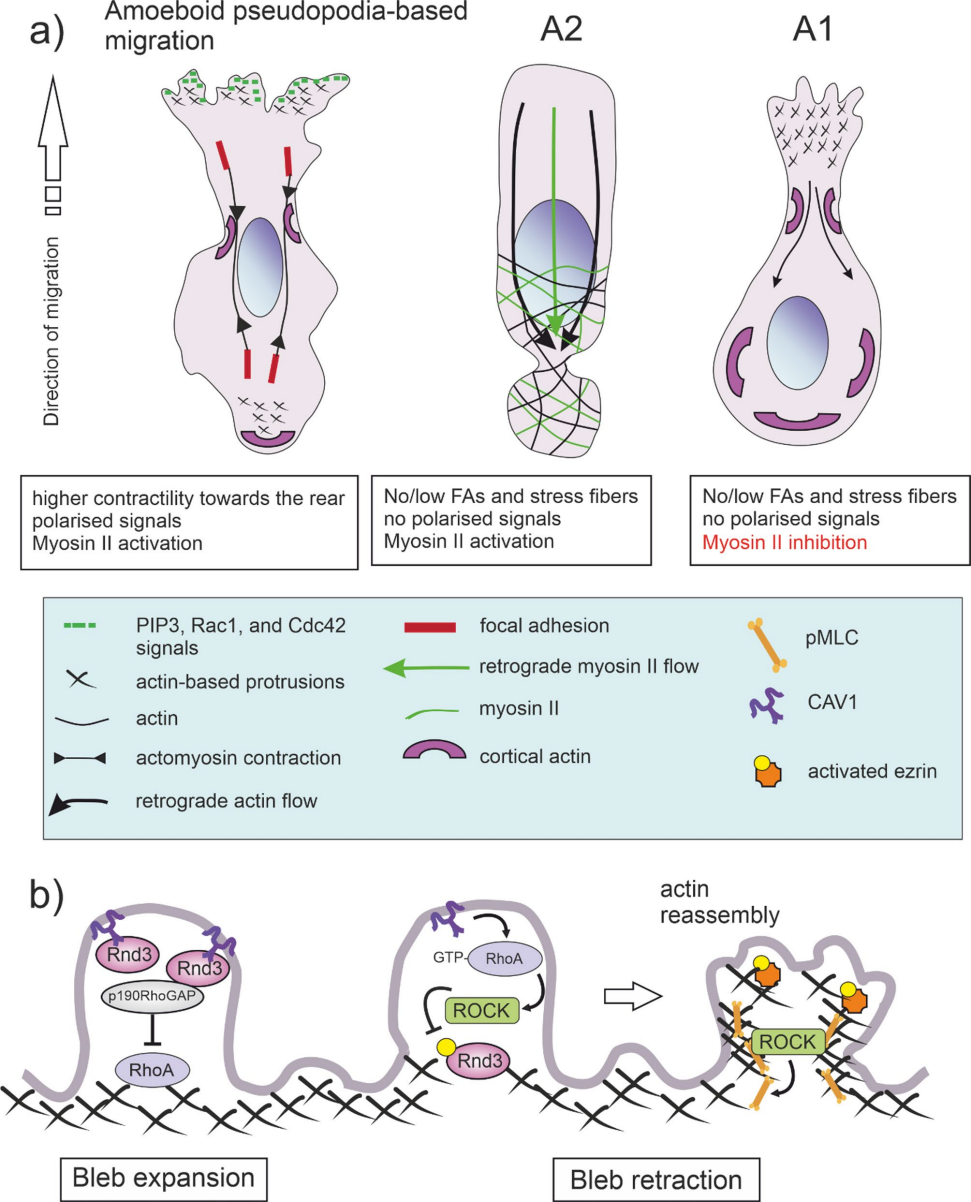
Amoeboid migration. **a** Amoeboid migration involves cells with the microtubule-organising centre located behind the nucleus. Unlike mesenchymal movement, it does not depend on polarisation and focal adhesions, except for pseudopod-based amoeboid migration using transient focal adhesions (not strong). In pseudopod-based amoeboid migration, cells are elongated and form actin-rich protrusions led by polarised Rac1, Cdc42, and PIP3. Two other subtypes of amoeboid migration exist: A1 cells are round with small protrusions and fast actin flow at the front, while A2 cells are ellipsoidal with a large leader bleb and strong actomyosin flow to the rear. Both rely on confinement rather than focal adhesion traction. Based on Wong et al. [129] **b** The double-negative feedback loop between RhoA and Rnd3 regulates the bleb cycle. Expansion Phase: Rnd3 localises to the bleb’s actin-free plasma membrane (PM), suppressing RhoA activity via the Rnd3-p190RhoGAP pathway. As the bleb expands, Rnd3 levels decrease, allowing RhoA activation. Retraction Phase: CAV1 regulate RhoA activity by inhibiting p190RhoGAP, which results in RhoA activation, or by directly affecting RhoA localisation within the PM, leading to its activation. Activated RhoA-ROCK phosphorylates Rnd3 (localised in the CAV1-rich fractions of lipid rafts), removing it from the PM and further activating RhoA. ROCK also phosphorylates ezrin. Phosphorylated myosin light chain (pMLC) is recruited to the actin cortex, driving bleb retraction through myosin II. Adapted from Ikenouchi et al. [117]

Amoeboid migration is characterised by a rounded cell morphology and low adhesion forces of migrating cells. This migration mode is named after the movement of the amoeba *Dictyostelium discoideum* [109]. Amoeboid cells migrate by adapting to their environment rather than remodelling it. In contrast with mesenchymal migration, amoeboid migration does not depend on the ability to form focal adhesions [108]. Although adhesion is not strictly required, amoeboid cells may still utilise weak or nonspecific surface interactions and pseudopodia to facilitate rapid movement (~ 10 μm/min)[110, 111]. Unlike in mesenchymal migration, pseudopodia-based amoeboid movement can proceed without strong ECM attachment. Pseudopodia—3D, actin-filled protrusions requiring WASP and SCAR proteins—are frequently used for navigation [110]. Inhibition of proteases or enhancement of actomyosin contractility often pushes cells toward amoeboid migration [112, 113]. Amoeboid cells can migrate using either high-contractility myosin II-dependent migration driven by membrane blebbing (A2-type), eventually leading to the formation of large “stable blebs” comprising most of the cell content, or protrusion-based migration (A1-type) that occurs under low cell contractility (Fig. 3A).

A2 migration is typically induced by physical confinement and relies on strong actomyosin contractility and retrograde flow, while A1 migration depends on local actin dynamics [108]. These processes are strongly ATP-dependent [114]. Interestingly, Liu et al.‘s findings show that confinement-induced amoeboid-like migration promotes rearward mitochondrial positioning to support polarised cytoskeletal dynamics, favouring a more energy-efficient mode compared to mesenchymal migration [115]. A2 cells migrate significantly faster (5.3 ± 1.5 μm/min) than A1 cells (1.7 ± 0.4 μm/min), and they both are much faster than mesenchymal cells (0.234 ± 0.09 μm/min)[108]. A2 amoeboid movement, also known as leader bleb-based migration, involves large, stable blebs that form at the front of the cell. These blebs form through the local detachment of the plasma membrane from the actin cortex, expand via cytoplasmic flow, and retract upon actin reassembly and myosin II-mediated contraction. Ezrin-rich, rigid uropod-like structures are also observable at the cell rear in amoeboid blebbing cells [116–118]. However, it is the localisation of RhoA activity and the direction of membrane flow, not the position of the uropod, that determines the direction of cell migration. RhoA-generated uropods are enriched in myosin, ezrin, moesin, β1-integrin, and PI(4,5)P2[118].

Leader bleb-based amoeboid migration does not involve Rac1 activity or actin polarisation at the leading edge and does not generate lamellipodia [118]. Elevated intracellular Ca²⁺ levels triggered by spatial confinement induce RhoA oscillations aligned with microtubule dynamics, creating a molecular clock that governs cell speed in confined spaces [51]. CAV1 regulates RhoA activity through two key mechanisms: (i) inhibiting p190RhoGAP, which results in RhoA activation, and (ii) directly affecting RhoA localisation within the plasma membrane, leading to its activation. Control of CAV1 over the RhoA/ROCK pathways regulates the number of stress fibres and actomyosin contractility [9, 119] and limits mitochondrial ROS [120]. Upon activation, RhoA-ROCK phosphorylates Rnd3, localised in the CAV1-rich fractions of lipid rafts [121]causing its dissociation from the plasma membrane and further promoting RhoA activation. ROCK also phosphorylates and activates ezrin, a linker between the plasma membrane and actin filaments (ezrin is essential for Src-CAV1 interactions [122]). Subsequently, myosin II is recruited to the actin cortex, driving bleb retraction [117] (Fig. 3b). The rounded cell shape requires actin polymerisation along the plasma membrane to stiffen and contract the cell cortex. These cortical actin dynamics are critically controlled by RhoA and its effector ROCK, generating cortical tension, stiffness, and the maintenance of round cell morphology [113]. Initiating migration by localised activation of RhoA led to a decrease in CAV1 fluorescence intensity at the plasma membrane, the formation of caveolin-rich vesicles, and an increase in endocytosis at the cell’s rear. This polarised endocytosis—a process disrupted upon RhoA or ROCK inhibition [123]—is driven by retrograde plasma membrane flow [118] (endocytosed vesicles traffic from the back to the front of the cell; on the other hand, the plasma membrane flows towards the back of the cell). Endocytosis and retrograde membrane flow support uropod formation, cell body contraction and rear-end retraction and are commonly observed in amoeboid cells, including T-lymphocytes [123]. During RhoA-driven migration, myosin IIA and filamentous actin polarise to the cell rear [118]. Inhibition of ROCK with Y27632 blocked myosin polarisation, reduced bleb formation, and decreased the proportion of A2-type migrating cells. Similarly, treatment with myosin II inhibitor blebbistatin abolished A2 migration across all tested cell types [108]. Consistently, CAV1 depletion via siRNA markedly reduces phosphorylated myosin light chain levels—a key indicator of myosin II activation [49]highlighting the essential role of CAV1 in supporting amoeboid contractility and bleb-based motility.

Amoeboid migration is commonly observed in several highly invasive and metastatic cancers, particularly those characterised by strong actomyosin contractility and high RhoA/ROCK signalling, such as gastric cancer [124]breast cancer, prostate cancer, and melanoma [125]. Cancer cells can invade nerves through perineural invasion (PNI), often using an amoeboid phenotype for rapid movement. Inhibiting ROCK shifts cells to a mesenchymal state, reducing nerve association, migration through confined spaces, and PNI in mouse models [126]. Tumour cells dynamically switch between mesenchymal and amoeboid migration based on microenvironmental cues. This transition is governed by a mutually inhibitory circuit between Rac1 and Rho/ROCK signalling. High Rac1 activity favours elongated, adhesive mesenchymal movement, while active Rho/ROCK drives rounded, bleb-based amoeboid migration. CAV1 influences this balance by modulating Rac1 deactivation and supporting RhoA signalling, thereby promoting amoeboid traits under conditions such as low matrix stiffness or high contractility. Cytokine signalling, including LIF/IL-6–JAK/STAT pathways, further reinforces Rho-ROCK activity in amoeboid cells, maintaining invasive potential [125].

### Lobopodia

Based on the shape and the elastic properties of the ECM, fibroblasts can switch between a classical mesenchymal lamellipodia-based migration and amoeboid-like lobopodia-mediated migration. In an environment characterised by linear elasticity, fibroblasts prefer lobopodia (cylindrical, blunt-ended protrusions that display numerous small membrane blebs). Lobopodia are driven by RhoA-dependent myosin II activity but need no polarised distribution of Rac, Cdc42, or PIP3. Lobopodial migration is defined by polarised myosin II contractility cooperating with vimentin filaments and the linker protein nesprin 3, which together pull the nucleus forward and generate compartmentalised intracellular pressure [127]. This results in the nucleus acting as a physical barrier that separates a high-pressure anterior region from a lower-pressure posterior compartment, a hallmark of lobopodial migration [128, 129]. Unlike amoeboid A2-type motility, which also relies on actomyosin contractility but lacks adhesive structures, lobopodia-mediated migration retains prominent actin stress fibres and FAs. This distinction suggests that while both modes are contractility-dependent, lobopodia maintain stronger adhesion to the ECM. Inhibition of RhoA or ROCK disrupts actomyosin contractility and causes the fibroblasts to switch from lamellipodia-based migration without affecting migration speed [130]. These findings highlight the central role of RhoA–ROCK signalling in regulating lobopodial motility. As previously discussed, CAV1 is a key regulator of the RhoA–ROCK pathway and can enhance actomyosin contractility [91, 119]. Therefore, CAV1 is likely to contribute to lobopodia-mediated migration by promoting the cytoskeletal and contractile dynamics required for this specialised form of motility.

In summary, CAV1 plays a pivotal role in managing the formation and dynamics of leader blebs by regulating membrane tension, endocytosis, cellular tension, and actomyosin networks through its role in the RhoA/ROCK signalling axis. It may also contribute to the plasma membrane remodelling during bleb formation and retraction. By coordinating bleb size and formation in response to mechanical forces [131]especially in environments with mechanical constraints, CAV1 enables cells to adapt to mechanically challenging environments. Beyond bleb regulation, CAV1’s ability to regulate actin dynamics, membrane tension, and intracellular signalling makes it a central player in coordinating the formation and function of various membrane protrusions crucial for migration, invasion, and adhesion, such as lamellipodia, filopodia, lobopodia, podosomes, invadopodia, and pseudopodia (see Table 1).

**Table 1:** Cav-1 function in regulating migration-associated membrane protrusions

	Structure	Dynamics based on	Membrane tension	CAV1 function
lamellipodia	broad, sheet-like	branched actin	high tension at the leading edge	mediates FAs turnover and Rac1 signalling[40, 53, 71]
filopodia	thin, finger-like	parallel actin bundles	high tension at the tips	CAV1 affects Cdc42 activity by modulating Src-dependent pathways[132, 133]
lobopodia	bulbous, blunt	RhoA-dependent myosin II activity	moderate tension	CAV1 regulates RhoA activity[91, 119]
pseudopodia	amorphous extensions of the cell membrane	crosslinked actin filaments	low tension, transient dynamics	CAV1 regulates FAs turnover, Cdc42, Rac1, and RhoA-ROCK signalling[40, 53, 71, 119]
podosomes	rings, belts, rosettes with degradative ability	Dense actin core surrounded by an actin-rich cloud	localised at adhesion sites	CAV1 regulates Src and Cdc42 activation and integrin-mediated adhesion required for podosome assembly[29, 30]
invadopodia	dot-shaped areas with degradative ability	dense actin filaments enriched in integrins	moderate tension	CAV1 enhances the recruitment of Src, cortactin, and MT1-MMP to invadopodia[26, 27, 55] and terminating Rac1 signalling by regulating its degradation[40, 53, 58].

## CAV1 in migration managed by the osmotic engine

**Fig. 4:**
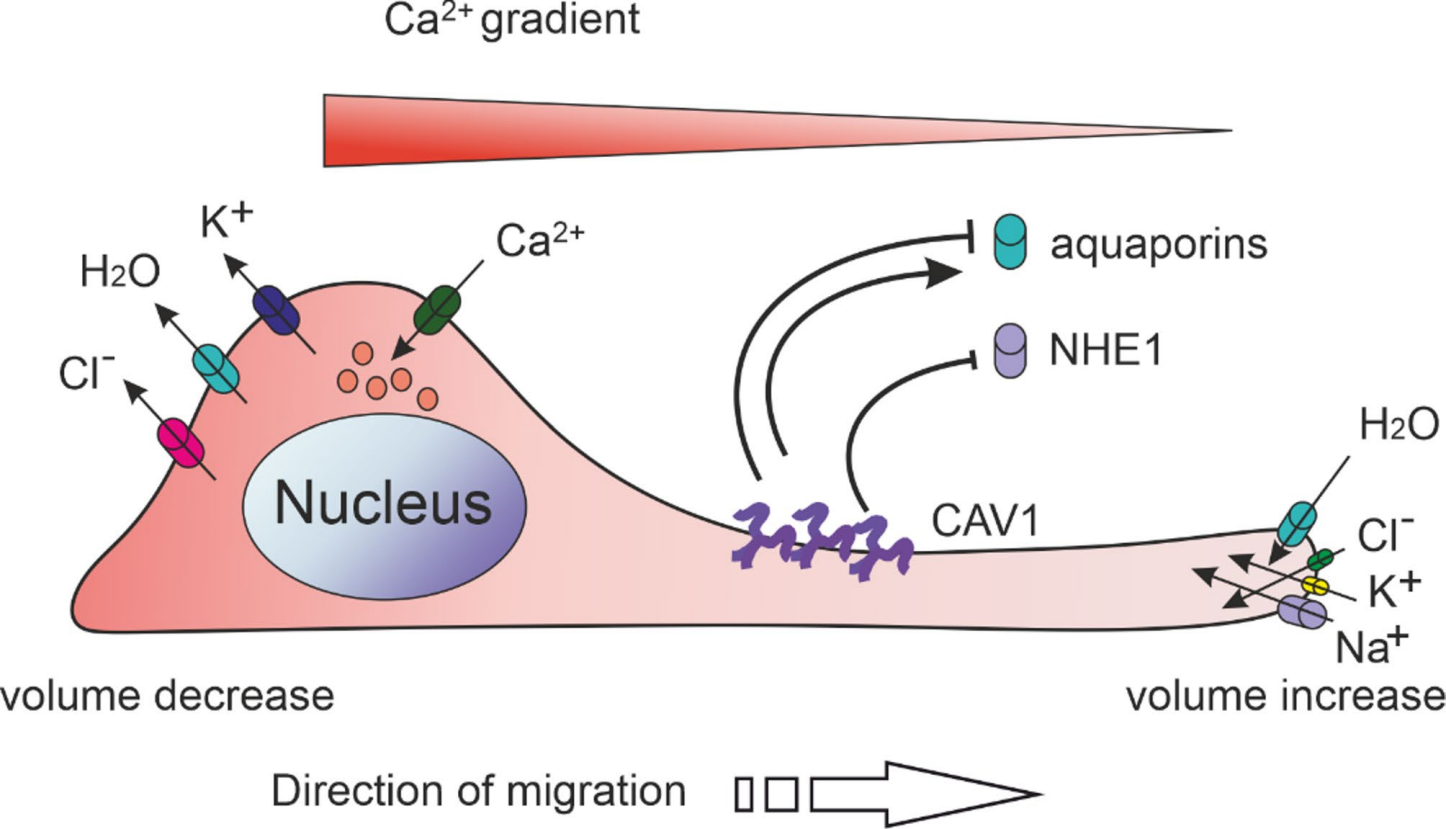
Osmotic engine. Osmotic engine-based migration occurs when cells are confined within rigid channels. Actomyosin contractility and focal adhesions are dispensable for this type of migration. NaCl and water uptake occur at the leading edge, contributing to volume gain, whereas KCl and water efflux lead to volume loss at the trailing edge. Ion and water channels such as NHE1 and aquaporins (AQPs) are polarised to the cell’s leading and trailing edges to facilitate specific water and ion fluxes. The intracellular Ca^2+^ gradient regulates the associated ion transporters. The red-to‐pink gradient depicts the high‐to‐low subcellular concentrations of Ca^2+^. Adapted from Morishita et al. [148]. CAV1 has been implicated in Ca^2+^ homeostasis [50] and regulates the activity and expression levels of NHE1 and AQPs

Migration through physically confined environments, such as rigid microchannels, can occur independently of actin or myosin II-mediated contractility [134]. According to the osmotic engine model, motility in these settings relies on polarised water influx and efflux at the leading and trailing edges, respectively (Fig. 4). While actomyosin contractility is dispensable, actin polymerisation is required for establishing the polarised localisation of aquaporins (AQPs) And ion transporters, such as sodium-hydrogen exchanger 1 (NHE1). Notably, inhibition of actin polymerisation, but not microtubule polymerisation, prevents NHE1 localisation to the leading edge of osmotically moving cells and suppresses this migration mode [135].

CAV1 emerges as a critical regulator in this context by controlling the localisation, trafficking, and expression of both AQPs and NHE1. NHE1 resides in CAV1- and cholesterol-enriched membrane microdomains, where CAV1 negatively regulates its activity [136]. This spatial regulation suggests that loss of CAV1 may enhance NHE1 function, supporting osmotic migration and promoting invasiveness. Similarly, CAV1 modulates the surface expression of several AQPs [137–139]which are key facilitators of water flux during osmotic engine-driven migration [135, 140–142]. CAV1 downregulation or loss has been associated with higher expression of AQP1[143] but decreased AQP4 expression [144]. In lung cancer cells, CAV1 knockdown disrupted hydrostatic pressure-induced AQP1 upregulation, suppressing ERK1/2-Akt1/2 signalling and reducing migration. Notably, elevated hydrostatic pressure had minimal impact on the migration and volume of normal epithelial cells. These findings reveal a novel mechanism linking high interstitial fluid pressure to cancer cell invasiveness [145].

Together, this evidence supports the potential of AQPs and NHE1 as downstream effectors of CAV1, positioning them as candidate biomarkers or therapeutic targets for tumours invading through confined spaces. For example, NHE1 expression is significantly elevated in breast cancer tissue compared to adjacent normal tissue and is also higher in doxorubicin-resistant cancer cells than in sensitive counterparts. Notably, this increased NHE1 expression can be therapeutically targeted with the specific inhibitor cariporide, which sensitises resistant breast cancer cells to doxorubicin [146]. AQPs contribute not only to tumour cell migration and angiogenesis but also to tumour-associated oedema in both solid and haematological malignancies. Given their roles in tumour progression, AQPs have emerged as promising therapeutic targets. Importantly, CAV1 regulates the expression, trafficking, and membrane localisation of several AQPs, linking its function to AQP-mediated processes. Thus, targeting AQPs, particularly in the context of CAV1 dysregulation, may offer novel strategies to disrupt tumour progression and metastasis. To gain a deeper understanding of the function of AQPs in cancer, as well as their significance as biomarkers and target molecules, please see Wang et al.‘s review article [147].

## CAV1 and collective cell migration

Collective cell migration allows groups of cells to migrate more effectively than individual cells. While single cells may migrate faster, their movement tends to be less persistent and lacks directional stability. Collective migration requires stable intercellular junctions and coordinated front–rear polarity across the group. This migration mode relies on actomyosin contractility to generate and resist forces and requires ECM remodelling. A feedback loop between phosphorylated CAV1 and contractile actin can support persistent cell migration [44]. CAV1 regulates the localisation of cathepsin B, pro-urokinase plasminogen activator (pro-uPA), and their receptors to caveolae, promoting ECM degradation and invasion. Downregulation of CAV1 in HCT 116 cells reduced collagen IV degradation and inhibited invasion [149].

In collective migration, specialised cells called leader cells (LCs) occupy the front of the migrating group, guiding follower cells behind them. In the endothelium, LCs form dynamic protrusions called VE-cadherin fingers engulfed by follower cells via VE-cadherin/catenin complexes and Arp2/3-mediated actin polymerisation [150]. Phosphorylation of CAV1 enhances its association with β- and γ-catenin, weakening their binding to VE-cadherin and enabling junction reorganisation [151]. The contractile actomyosin cable prevents the initiation of new LCs [152]. Consequently, ROCK and myosin-II inhibition triggered LC formation in colorectal cancer [153]. Key functions of LCs include pathfinding or path generating, coordinating with nearby cells, and enhancing the survival of the follower cells [154, 155]. Leader cell behaviour is driven by mechanical and biochemical cues, often regulated by small GTPases, such as RhoA, Rac, Cdc42 [152, 156], RhoGEFs (e.g. β-Pix [157] or Par3/6[158]), and adhesion receptors (e.g. integrin β1[159, 160]). Many of these factors are linked to CAV1 functions (as discussed earlier in the manuscript). For example, reciprocal regulation between β1 integrin and CAV1 is assumed [88]. Additionally, the tension on collagen I fibres contributes to the YAP-TEAD-driven transcriptional program in LCs [161]. CAV1 regulates YAP mechanotransduction via actin control in response to substrate stiffness [162] and caveolae enhance YAP/TAZ responses to shear stress. Interestingly, both CAV1 and CAVIN1 are direct YAP/TAZ-TEAD target genes, and loss of caveolae leads to YAP/TAZ hyperactivation [163].

LCs are highly polarised in the direction of the migration, exhibiting an elongated shape with dynamic actin-based protrusions like filopodia and lamellipodia. The pulling forces of LCs trigger the translocation of Merlin (NF2; a key contact inhibition regulator) from cell-cell contacts to the cytoplasm, driving polarised Rac1 activation and lamellipodium formation [164]. Interestingly, pull-down experiments showed that NF2 can bind to and activate CAV1[165]. LCs express distinct protein profiles that regulate cell-cell adhesion, promote partial EMT, restrict leadership to LCs, and enable tissue proteolysis during the collective invasion. Active Rac, integrin β1, and PI3K were localised at the leading edge of LCs but not in followers. Inhibiting these molecules disrupted collective migration [159]. All these molecules (Rac, integrin β1, and PI3K) localised at the leading edge of LCs can be influenced by CAV1. PI3K activity can be triggered by mechanosensitive CAV1 activation [27]. CAV1 also regulates Rac1 by promoting its activation via Rab5[54] and Src localisation [55] and its deactivation through Csk-mediated Src inactivation [56] and Rac1 degradation [40]. Furthermore, CAV1 in caveolae keeps ERK inactive, but under stretch stress, it translocates to non-caveolar sites to activate ERK via β1 integrins, Fyn, and Shc [46]. ERK activation triggers cell contraction, pulling neighbouring cells and inducing their ERK activation. Front-rear polarisation ensures unidirectional ERK waves, aligning cell polarity and driving coordinated, long-range migration [166]. CAV1 depletion decreased collective epithelial motility. Mechanistically, CAV1 knockdown increases PI(4,5)P2 at the plasma membrane, recruiting FMNL2 to stabilise junctional F-actin. FMNL2 downregulation in CAV1 knockdown cells restores collective epithelial motility [167].

Due to their role in generating mechanical forces required for migration through the ECM, LCs likely have higher energy demands than follower cells. The energy levels of LCs (as revealed by the intracellular ATP/ADP ratio) must be high to exceed a threshold for invasion. As ATP is depleted during migration, exhausted LCs are eventually replaced by more energetically viable follower cells. When put into a denser collagen matrix, LCs’ durability decreases [168]. Several studies also showed that functional mitochondrial oxidative phosphorylation (OXPHOS) is critical for cells to secure metastatic spreading. Pharmacological inhibition of pyruvate dehydrogenase and dihydroorotate dehydrogenase (DHODH), or removal of mitochondrial DNA, reduced the ability of cancer cells to invade [169–171]. Consequently, functional mitochondria are probably necessary for LCs’ activity and collective migration. Mitochondria localise at the leading edge of migrating cancer cells to serve as local energy sources [172, 173]. Miro1-mediated mitochondrial positioning at the leading edge boosts ATP supply, supporting actin dynamics, membrane protrusion, and focal adhesion stability. In contrast, loss of mitochondrial RhoGTPase Miro1 disrupts these processes, slowing collective and single-cell migration [173, 174].

Cell migration induces mild mitochondrial damage, with increased mitochondrial ROS production and activation of the mitochondrial unfolded protein response associated with highly metastatic cancer cell phenotypes [175, 176]. In cancer cells, CAV1 phosphorylation prevents basal mitophagy, resulting in the accumulation of damaged mitochondria [120]. In contrast, CAV1 depletion or dephosphorylation releases Mfn2 and Drp1, promoting mitochondrial fusion, fission, and mitophagy initiation [177]. While slightly damaged mitochondria do not threaten the cancer cell, severely damaged mitochondria can trigger cell death. Since CAV1 phosphorylation prevents basal mitophagy, CAV1-positive tumour cells must employ alternative mechanisms to eliminate irreparably damaged mitochondria. During migration, one such mechanism involves the packaging and discarding of damaged mitochondria into vesicular structures called migrasomes [178, 179]. This intraluminal vesicle transport to migrasomes is mediated by Rab10 and CAV1. However, the exact role of CAV1 in migrasome cargo trafficking remains unclear. Anyway, the intraluminal vesicle transport to migrasomes is independent of CAV1 function in caveolae [180]. Li et al. showed that migrasomes contained significantly higher levels of CAV1 compared to extracellular vesicles derived from L929 fibroblast cells [180]. Through migrasomes, migrating cells release damaged mitochondria into the surrounding environment, which can have serious consequences. Immune cells like macrophages or T-cells can internalise extracellular vesicles/migrasomes containing damaged mitochondria. Cancer cell mitochondria are often coated with USP30, preventing their degradation. This allows them to replace T-cell mitochondria, inducing T-cell senescence and impairing their cancer-killing ability [181, 182].

## CAV1 and metastasis

Cell migration’s molecular mechanisms and metastatic disease progression are highly correlated [19, 183]. CAV1 is expected to be involved in metastasis based on its role in migrating cells. Indeed, in a syngeneic 4T1 model, CAV1 knockout reduced lung metastasis of breast cancer by impairing integrin α3 regulation. CAV1 knockout also reduced motility, MMPs production, and extracellular vesicle secretion in 4T1 cells [106]. Similarly, CAV1 enhanced cell invasion by promoting filopodia formation in lung adenocarcinoma [133]. CAV1-mediated activation of Src/Akt signalling was required for CAV1-enhanced migration, as inhibition of glycolysis by 2-deoxy-D-glucose or disruption of Src/Akt signalling reduced CAV1-mediated lung metastasis of B16-F10 cells [184]. Inflammatory breast cancer models have demonstrated that CAV1 overexpression activates Akt1, which in turn phosphorylates RhoC GTPase [185]. CAV1 has also been associated with increased lymph node metastasis [186] or brain metastasis of non-small cell lung cancer [187]. CAV1 promotes hepatocellular carcinoma progression and metastasis through the Wnt/β-Catenin pathway [188] and increases the number of Ewing sarcoma metastases by regulating MMP-9 via the MAPK/ERK pathway [189]. High CAV1 expression levels regulated by FoxM1 correlated with metastatic potential and EMT in pancreatic cancer, while CAV1 knockdown inhibited these processes [190]. In renal cell carcinoma, CAV1 promotes invasion and, alongside pERK, predicts metastases in patients with localised disease [191]. Similarly, high CAV1 expression was also linked to metastasis and poor prognosis in clear cell renal cell carcinoma [192]. Although CAV1 suppresses primary tumour growth in melanoma, it promotes lung metastasis. CAV1 expression increases with melanoma progression and peaks at the metastatic stage. In a post-operative setting, increased CAV1 expression favoured the development of lung metastasis, suggesting its role as a risk factor for postoperative metastasis [193]. The pro-metastatic effect of CAV1 in melanoma depends on its phosphorylation at tyrosine 14 and ECM–integrin interactions. The non-phosphorylatable Y14F mutation reduces metastasis without affecting the tumour suppressor function of CAV1, making Y14 phosphorylation a promising target to block metastasis while preserving the beneficial properties of CAV1[194]. In hepatocellular carcinoma (HCC), CAV1 promotes tumour growth and metastasis via autophagy inhibition [195] or angiogenesis [196]. Hypoxia-induced CAV1 can trigger HCC metastasis via S100P activation through the NF-κB pathway. Silencing CAV1 or NF-κB reduced S100P expression and cell migration, while S100P restoration rescued metastatic potential [197]. CAV1 is also a metastasis-related gene and a candidate androgen-resistance gene for prostate cancer [198].

Metastatic cancer cells must resist anoikis to survive fluid shear stress in the circulatory and lymphatic systems. Li et al. demonstrated that low shear stress (2 dyn/cm^2^) increased anoikis resistance in MDA-MB-231 breast cancer cells due to CAV1 overexpression, while CAV1 depletion restored their sensitivity to anoikis [199]. CAV1 also enhanced anoikis resistance in MCF-7 human breast cancer cells, but decreased the proliferation rate of MCF-7 cells and significantly reduced their ability to form colonies [200]. Silencing fatty acid synthase (FASN) and estrogen receptor α (ERα) suppressed breast cancer cell growth, but increased invasion via a CAV1-dependent mechanism [201]. Surprisingly, the complete loss of CAV1 in a transgenic mouse model of breast cancer accelerated both tumourigenesis and metastasis [202]. In gastric cancer cells, CAV1 promotes anoikis resistance by regulating Src-dependent EGFR-ITGB1 signalling [203]. Similarly, in human non-small cell lung cancer, elevated CAV1 expression correlated with anoikis resistance [204].

Lipid rafts are cholesterol- and sphingolipid-rich membrane microdomains that organise signalling in cancer. CAV1 is a key raft-associated scaffolding protein and plays a central role in maintaining raft integrity and function. Within these microdomains, raft compartmentalisation facilitates transmembrane receptor clustering, protects signalling molecules from degradation, and serves as a platform to amplify intracellular signalling cascades. Through these mechanisms, CAV1 and lipid rafts regulate key processes such as adhesion, migration, EMT, and cell survival, making them important contributors to metastasis [205]. For example, CAV1 in lipid rafts regulates HER2 receptor trafficking and the effectiveness of HER2-targeted therapies, as co-localisation of CAV1 with anti-HER2 antibody-drug conjugates (ADCs; e.g. trastuzumab, trastuzumab-emtansine (T-DM1), trastuzumab-deruxtecan) can impair lysosomal degradation of HER2-ADC complexes, reducing treatment efficacy. These findings have led to the identification of CAV1 as a possible predictive biomarker of response to HER2-targeted therapies [206] and the potential of using CAV1-PET and optical imaging for detecting gastric tumours [207]. Disruption of lipid rafts by cholesterol-lowering agents or cholera toxin can decrease HER2 dimerisation and recycling, increase membrane HER2 levels, and enhance the binding and potency of HER2-targeting antibodies and ADCs [206]. Disruption of lipid rafts by MβCD led to downregulation of CAV1, LRP6, β-catenin, survivin, caspase-3, Bcl-2, and Bax. In contrast, cholesterol supplementation restored CAV1 expression and increased the clonogenic potential of the triple-negative breast cancer cells [208].

Lipid rafts are sensitive to environmental pollutants. Environmental toxins can significantly impact the structure and function of lipid rafts (including caveolae), potentially disrupting cell signalling pathways and contributing to cancer metastasis. Pesticides (DDT and lindane), heavy metals (cadmium and mercury), and polycyclic aromatic hydrocarbons disrupt lipid profiles, cholesterol metabolism, lipid-protein interactions, and the raft structure by integrating into the membrane. For example, Bisphenol A (BPA) and cadmium promote cancer cell migration through integrin signalling. BPA enhances integrin/FAK/ERK signalling, driving cytoskeletal changes and cell motility, contributing to tumour invasion. Similarly, cadmium activates integrins and FAK, stimulates Rac1, and stabilises β-catenin via GSK3β inhibition, promoting Wnt signalling and metastasis in triple-negative breast cancer [209].

### Caveolae-independent functions of CAV1

Although CAV1 is best known as a structural component of caveolae, it also plays crucial roles outside these plasma membrane invaginations. CAV1 localises to intracellular organelles such as Golgi apparatus, mitochondria, nuclei, and the endoplasmic reticulum (ER), enabling responses to extracellular signals [210]. CAV1 is also enriched in mitochondria-associated ER membranes (MAMs), which are contact sites between the ER and mitochondria, important for Ca^2+^ and lipid homeostasis [211]. CAV1 knockout liver cells show reduced ER–mitochondria contacts, leading to cholesterol accumulation in MAMs [211]. On the other hand, in cancer cells, during early ER stress, DRP1 relocates to the ER, while its PKA-phosphorylated form accumulates at MAMs, enhanced ER–mitochondria contact, promoting mitochondrial elongation and ER expansion. By inhibiting PKA, CAV1 reduces pDRP1 levels and thereby affects mitochondrial function [212]. The distance between the ER and mitochondria is crucial for effective mitochondrial calcium uptake and optimal oxidative metabolism [213]. Consequently, MAM proteins, including CAV1, influence cell migration and cancer progression through mitochondrial calcium dynamics [214]. Cells with CAV1 overexpression or knockdown exhibit altered mitochondrial morphology, OXPHOS activity, ER–mitochondria spacing, and lysosomal function [215]. CAV1 also regulates mitophagy [177] and is involved in regulating mitochondrial cholesterol levels. In the absence of CAV1, cholesterol accumulates in mitochondrial membranes, leading to organelle dysfunction and significant metabolic consequences for the cell [216]. Non-caveolar CAV1 in prostate cancer cells can upregulate VEGF-A expression, which in turn stimulates the proliferation, migration, and tube formation of lymphatic endothelial cells (LECs). This process is crucial for lymphatic metastasis, a hallmark of prostate cancer progression [217].

CAV1 facilitates the efflux of cholesterol [218]CAV1 may help direct LDL-derived cholesterol from intracellular sites back to the cell surface. In fibroblasts, caveolin overexpression increases cholesterol efflux, reduces S-phase cholesterol buildup, and delays mitotic entry, linking cholesterol handling to cell cycle control [219]. Moreover, CAV1 supports the delivery of lipids to intracellular organelles and regulates lipid droplet formation [220]. This lipid-transporting function is critical for maintaining membrane fluidity, organising receptor localisation, and modulating responses to mechanical stress [206, 221, 222]. In cancer, dysregulated CAV1 expression can profoundly alter lipid metabolism, which is a hallmark of malignancy [223]. In aggressive tumours, CAV1 overexpression is often associated with lipid droplet enrichment, promoting energy storage and membrane biogenesis necessary for rapid proliferation and migration [224].

CAV1 also plays an important role in the biogenesis and function of extracellular vesicles (EVs), particularly in cargo sorting and vesicle composition. As a scaffolding protein with membrane-organising capabilities, CAV1 is enriched in specific microdomains of the plasma membrane and endosomal compartments, which are involved in the formation of EVs. Additionally, CAV1 can affect the biophysical properties of vesicle membranes through its interactions with cholesterol and sphingolipids, which are important for the stability, targeting, and uptake of EVs [225].

### CAV1 in stromal interactions

The tumour stroma plays a key role in modulating invasion modes during cancer progression. Tumour cells migrating through compliant, deformable ECM often adopt an amoeboid phenotype, characterised by actomyosin contractility and microvesicle release, whereas stiffer matrices promote invadopodia formation and mesenchymal motility. Cancer-associated fibroblasts (CAFs) can further influence this plasticity by promoting epithelial-to-mesenchymal transition (EMT) and recruiting endothelial progenitor cells to support a shift from mesenchymal to amoeboid transition (MAT). This switch is often mediated by the RhoA/ROCK1/MLC-P pathway, triggered by stromal cues like plasminogen activator inhibitor-1 or cytokines, such as IL-6 and IL-8, secreted by bone marrow-derived mesenchymal stem cells that have adopted a CAF-like phenotype [20]. In melanoma, amoeboid cells also secrete high levels of TGFβ, which sustains contractility via the SMAD2–CITED1 transcriptional program [226]. As TGFβ is a key activator of CAFs [227]amoeboid cancer cells can support CAF activation. In prostate cancer, the neural microenvironment contributes to tumour progression through a unique paracrine mechanism involving CAV1. Stromal and perineurial cells upregulate and secrete CAV1 in response to TGFβ1 produced by adjacent tumour cells, which then use this extracellular CAV1 to inhibit apoptosis and enhance survival, particularly during perineural invasion. Neutralising CAV1 can partially reverse this effect [228].

Stromal components, including fibroblasts, endothelial cells, and immune cells, significantly impact tumour growth and metastasis. Interactions between tumour cells and the surrounding stroma shape tumour phenotype, with cancer cells often inducing a reactive stroma that promotes invasion. Although many stromal cells express CAV1, its role in tumour-stroma crosstalk remains elusive and is likely to be highly context-dependent. In several cancer types, including breast and prostate cancer, the loss of CAV1 expression in stromal cells is associated with increased metastasis [229–231]. High levels of CAV1 in the stromal tissue surrounding the tumour, rather than within tumour cells, were associated with reduced metastasis and improved survival in breast cancer patients [229]. In metastasis-associated macrophages, CAV1 loss promoted lung metastasis by enhancing angiogenesis [232]. Reduced stromal CAV1 marks poor prognosis and aggressive melanoma metastasis [233]. One of the presumed mechanisms looks as follows: cancer cells produce reactive oxygen species (ROS) that spread to nearby stromal cells, including fibroblasts. This oxidative stress activates key transcription factors such as HIF-1α and NF-κB in CAFs, pushing them toward aerobic glycolysis. At the same time, ROS downregulate CAV1, contributing to mitochondrial dysfunction in CAFs. This metabolic shift leads CAFs to produce energy-rich metabolites like lactate and pyruvate, which cancer cells then use to fuel their growth and invasion (reverse Warburg effect) [234]. As part of the reverse Warburg effect, cancer cells activate TGFβ and inflammatory signalling in CAFs, further promoting glycolysis. TGFβ signalling drives this catabolic shift in CAFs, independent of its source (cancer or stromal cells). TGFβ activates fibroblasts via autocrine or paracrine signalling, promoting their metabolic reprogramming. In contrast, TGFβ signalling in cancer cells alone has minimal impact on tumour growth [235]. CAV1-deficient fibroblasts secrete pro-tumorigenic factors (e.g., VEGF, MIP-2, IL-6), promoting metastasis [236]. Loss of CAV1 also upregulates PAI-1/2, likely via oxidative stress [237]. PAI-1 is among the most reliable biomarkers in many cancer types [238].

On the other hand, in some carcinomas, CAFs and cancer cells exhibit elevated CAV1 expression. High CAV1 levels may enhance contractility of CAFs or cancer cells and their directional migration, facilitating extracellular matrix remodelling and tumour cell invasion. This prometastatic effect is potentially mediated via RhoA-driven contractility and CAV1-dependent regulation of p190RhoGAP, contributing to a mechanically supportive and invasive stromal microenvironment [239]. CAV1 expression in the tumour stroma varies depending on tumour stage, microenvironment, and fibroblast subtype. In early or invasive tumours, high CAV1 levels in CAFs and cancer cells promote contractility, matrix remodelling, and invasion, often via RhoA signalling. In contrast, in advanced tumours or oxidative environments, stromal CAV1 is downregulated due to stress signals like TGFβ, promoting metabolic reprogramming and tumour support via the reverse Warburg effect [235]. This dynamic regulation reflects the stromal plasticity and adaptation to local cues.

CAV1 functions not only as a structural and signalling protein but also as a secreted molecule incorporated into extracellular vesicles (EVs), thereby exerting systemic effects. Mechanical stretch increased CAV1 trafficking into EVs and their release [240]. Secreted CAV1 promoted cell survival, clonal expansion, and metastasis in androgen-insensitive prostate cancer [241]. CAV1 present in tumour-derived exosomes (TDEs) serves as a key driver of cancer stem cell (CSC) traits and EMT in prostate cancer undergoing neuroendocrine differentiation, primarily through activation of the NFκB signalling pathway. Additionally, exosomal CAV1 from metastatic castration-resistant prostate cancer cells can promote resistance to both radiation and chemotherapy in recipient cells [242]. Motile hepatocellular carcinoma (HCC) cells secrete EVs that significantly enhance non-motile cells’ migratory and invasive capabilities. These EVs derived from metastatic cell lines were enriched for protumourigenic RNAs and proteins, including the MET proto-oncogene, S100 family members, and caveolins. The uptake of these EV cargoes activated the PI3K/AKT and MAPK signalling pathways, leading to increased secretion of active MMP-2 and MMP-9[243]. Under acidic conditions, CAV1 associated with melanoma EVs was more efficiently transferred to less aggressive tumour cells [244]. Notably, CAV1-containing EVs can transfer malignant traits to recipient cells. Several metastasis-related proteins, including tenascin (TnC), cysteine-rich Angiogenic inducer 61 (Cyr61), and S100 proteins, were detected exclusively in CAV1-containing EVs [245]. Breast cancer-derived EVs deliver CAV1 to distant metastatic sites, contributing to pre-metastatic niche formation in the lungs. CAV1 induces inflammatory chemokines in lung epithelial cells and promotes TnC secretion from lung fibroblasts, facilitating ECM remodelling [246]. Additionally, it drives M2 macrophage polarisation and angiogenesis via the PTEN/CCL2/VEGF-A pathway [247]. CAV1 regulates EV biogenesis and protein cargo sorting (such as TnC) by governing cholesterol homeostasis in multivesicular bodies, effectively acting as a cholesterol rheostat to govern tumour–stroma communication [248]. In chemoresistant gastric cancer cells, CAV1-mediated endocytosis is essential for cell survival, and CAV1 downregulation induces cell death [249]. Furthermore, CAV1 mediates Laminin β2 sorting into EVs derived from hypoxia-resistant gastric cancer cells, promoting peritoneal metastasis in normoxic cells via Akt activation. Rho-associated kinase 1 phosphorylates CAV1 at Y14, enhancing Laminin β2 sorting through Rab11 activation. Combined plasma EV-associated CAV1 and Laminin β2 levels are predictive biomarkers for peritoneal metastasis in gastric cancer [250].

Migrasomes, vesicular organelles formed on retraction fibres of migrating cells, mediate intercellular communication through migracytosis. Li et al. identified CAV1 and Rab10 as markers of intraluminal vesicles within migrasomes. Colony-stimulating factor-1 (CSF-1) is transported to migrasomes via Rab10-CAV1-mediated vesicle trafficking, and functional protein CSF-1 can be released via this pathway [180]. CSF-1 can potentiate tumourigenicity and invasiveness, and accelerate metastasis [251, 252]. Another class of EVs containing CAV1 are large oncosomes (1–10 μm). These EVs originate from plasma membrane budding, particularly in highly migratory and aggressive tumour cells with an amoeboid phenotype. Large oncosomes facilitate the transfer of oncogenic signals, such as MMPs, RNAs, CAV1, and ARF6, contributing to enhanced migration of CAFs and cancer progression [253, 254]. In addition, prostate cancer cells secrete prostasomes enriched in CAV1 and MAL, contributing to prostate tumour signalling and intercellular communication [255].

Recent studies have revealed that mitochondria can be actively transferred within the tumour microenvironment. In many cases, tumour cells acquire mitochondria from surrounding cells in the microenvironment to enhance their own invasive and metastatic capabilities. Among the various mechanisms, tunnelling nanotubes (TNTs) appear to be the primary route for mitochondrial exchange between cells [256]. TNTs connecting neighbouring cells have been shown to contain significantly lower concentrations of CAV1 and Cavin-1 compared to the plasma membrane [257]. It can be hypothesised that CAV1 protects cells in the TME from extensive expansion of TNTs by stiffening the plasma membrane [258]and a decrease in stromal expression of CAV1 may lead to increased TNT production, enhancing the aggressiveness of neighbouring cancer cells.

### CAV1 as a prognostic biomarker and its potential for therapeutic targeting

CAV1 expression and localisation have significant prognostic and diagnostic value, according to several meta-analyses. Notably, high CAV1 expression in tumour epithelial cells was associated with worse prognosis and reduced overall survival in oesophageal, pancreatic, and hepatocellular carcinoma (HCC). However, this association was not evident in gastric or colorectal cancer, underscoring the tissue-specific roles of CAV1[100]. In prostate cancer, CAV1 is consistently overexpressed in tumour cells, serving as a risk factor and marker of poor clinical outcome, particularly in patients undergoing radical prostatectomy [102]. Low stromal CAV1 expression correlates with poor prognosis in breast and colorectal cancer [100, 259, 260]. These data collectively support the notion that CAV1 expression must be evaluated separately in tumour and stromal compartments. Such compartment-specific assessments could enhance the clinical utility of CAV1 as a predictive biomarker and guide personalised treatment strategies.

Given its central role in modulating cancer cell migration, invasion, therapy resistance, and communication with the microenvironment, CAV1 represents a compelling therapeutic target, especially in advanced cancers where its pro-metastatic functions dominate. Despite its established role in cancer progression, CAV1 cannot currently be targeted directly with specific therapies, due to several inherent challenges. As a scaffolding protein embedded within the plasma membrane and caveolae, CAV1 lacks the conventional enzymatic activity or surface-exposed ligand-binding domains typically exploited by small-molecule drugs or antibodies. Furthermore, its dual role as both a tumour suppressor and promoter, depending on cancer type, stage, and subcellular localisation, complicates direct therapeutic intervention. Instead, indirect targeting strategies targeting its regulators or effectors have emerged as simpler approaches. For example, inhibition of Src activity in MDA-MB-231 breast cancer and B16F10 murine melanoma cells using the Src family kinase inhibitor PP2 or dasatinib reduced CAV1 phosphorylation at Y14 and cell migration [72, 261]. Another approach involves activating the potassium channel Kv11.1 using NS1643 or PD115087, which triggers calcium-dependent activation of PTP1B. This leads to CAV1 dephosphorylation at Y14, promoting β-catenin–mediated cell–cell adhesion and reduced focal adhesion dynamics and motility [262].

Lipid rafts are closely linked to cancer progression and are significantly enriched in cancer stem cells (CSCs) compared to non-stem cancer cells. Key CSC markers like CD24, CD44, and CD133 localise to these rafts, which are essential for regulating CSC self-renewal, EMT, drug resistance, and interactions with the tumour niche. Disrupting lipid rafts with agents such as statins, alkyl phospholipids, and natural compounds like celastrol or emodin impairs raft integrity, reduces CSC stemness, and enhances therapy response, highlighting lipid rafts as critical targets for anti-cancer strategies [263]. One of the promising targetable downstream effectors of CAV1 is ROCK. CAV1 promotes the activation of RhoA–ROCK signalling, thereby enhancing cytoskeletal tension and invasive behaviour in cancer cells. Inhibiting ROCK activity has been shown to reduce migration, invasion, and metastasis in various tumour models, making it a promising strategy to indirectly target CAV1-driven motility [264].

## Conclusions and future perspectives

CAV1 plays a multifaceted role in cancer cell migration and metastasis, acting as a tumour suppressor in early-stage cancers and a promoter of invasion and metastasis in advanced disease. Through its regulation of key migratory signalling pathways and cytoskeletal dynamics, CAV1 emerges as a central orchestrator of cancer cell motility. In addition, CAV1-mediated EV biogenesis and extracellular matrix remodelling further contribute to metastatic dissemination by shaping the tumour microenvironment and the pre-metastatic niche. Despite substantial progress in understanding the context-dependent functions of CAV1, challenges remain in therapeutically targeting its dual roles. Future studies should focus on identifying the molecular determinants that control its switch from a tumour suppressor to a metastasis promoter. Understanding CAV1-associated signalling networks, including extracellular vesicle signalling, may offer novel therapeutic strategies to disrupt cancer cell migration and metastasis and prevent metastatic progression.

For example, pY14-CAV1 represents a compelling therapeutic target. This phosphorylation event is catalysed primarily by Src family kinases, which have broad cellular roles and are already targeted by several existing inhibitors (e.g., dasatinib, saracatinib). Furthermore, inhibition of Src activity in MDA-MB-231 cells using the Src family kinase inhibitor PP2 reduced CAV1 phosphorylation at Y14 and cell migration [72]. However, these inhibitors are not selective for pY14-CAV1, and systemic Src inhibition can result in off-target toxicity. To our knowledge, no small molecules have been developed that specifically block CAV1 Y14 phosphorylation. A more feasible strategy may involve disrupting pY14-CAV1 interactions with specific binding partners (such as focal adhesion or cytoskeletal regulators) or blocking its localisation to regions critical for signalling. Developing selective inhibitors or mimetics that interfere with the pY14-CAV1 interactome, without globally inhibiting Src, may provide a more targeted therapeutic window. This remains an area of high interest and unmet need. Future drug development could explore several promising avenues, such as peptidomimetics or small molecules that block the binding interface between pY14-CAV1 and its downstream effectors (e.g., proteins with SH2 domains involved in cytoskeletal remodelling or focal adhesion signalling). Allosteric inhibitors that prevent CAV1 conformational states required for Y14 phosphorylation or effector recruitment. Structural studies could guide the design of such molecules. Proteolysis Targeting Chimeras (PROTAC)-based degradation strategies targeting pY14-CAV1 selectively, assuming phosphorylation-specific binders can also be developed.

While our study focuses on the α isoform due to its unique Y14 phosphorylation site, the β CAV1 isoform, which lacks this region, may still contribute to cellular processes relevant to migration and metastasis through Y14-independent mechanisms. For example, α-CAV1 is preferentially localised in deep caveolae, while β-CAV1 is present in both deep and shallow forms, suggesting a molecular distinction between caveolar subtypes. Functional studies in HepG2 cells show that α-CAV1 efficiently induces caveolae formation, whereas β-CAV1 does so poorly. Co-expression of both isoforms or of CAV1 with CAV2 enhances deep caveolae formation, indicating cooperative roles [4]. Furthermore, the isoforms show distinct interactions with signalling pathways. Both BMP and EGF stimulation led to a redistribution of CAV1 isoforms on the cell surface, with β-CAV1 relocating into α-enriched domains. BMP receptors (BRIa and BRII) dynamically associate with both isoforms, but their activation by BMP-2 prompts redistribution, particularly toward α-CAV1 domains. Importantly, overexpression of β-CAV1 inhibits BMP signalling, whereas α-CAV1 does not [265, 266]. These findings suggest that α and β isoforms of CAV1 have distinct roles in caveolar structure and signalling regulation, with β-CAV1 potentially acting as a negative regulator of BMP-mediated pathways. This is important because BMP2 is highly overexpressed in non-small cell lung cancer (NSCLC), particularly in patients with lymph node metastases, and is associated with advanced tumour stage and metastatic burden. Using an orthotopic mouse model, it was demonstrated that BMP2 promotes lung adenocarcinoma metastasis through activation of the SMAD1/5/8 pathway, independent of KRAS signalling. Inhibiting SMAD1/5/8, either genetically or pharmacologically (e.g., with LDN193189), suppressed migration [267]. Further studies are needed to clarify whether β-CAV1 plays a compensatory, suppressive, or context-specific role in migratory behaviour.

Additionally, further research into CAV1-interacting molecules, such as CAV2, is essential for a comprehensive understanding of caveolae biology and its implications in cancer. CAV2, often co-expressed and forming hetero-oligomers with CAV1, plays a critical yet underexplored role in caveolae function and stability. While CAV1 is essential for caveolae formation, CAV2 modulates their structural integrity and signalling capacity. Interestingly, CAV2 has activities, opposing CAV1 in the regulation of some cellular processes, such as angiogenesis, endocytosis, and regulation of inflammatory responses [268]. Emerging evidence shows that CAV2 expression is upregulated in certain cancers and can promote invasion and metastasis [269–272]raising questions about its independent or synergistic roles in tumour progression. Given its potential impact on caveolae dynamics and metastasis, further research is needed to determine how CAV2 contributes to cancer cell behaviour and whether it modifies or enhances CAV1-dependent functions. Key questions include: Can CAV2 compensate for CAV1 loss or dysfunction? Does CAV2 influence EV cargo composition? And how does its expression affect cancer prognosis and therapy response? Investigating these interactions could uncover novel regulatory mechanisms that control caveolar function, cell migration, or therapy resistance. Moreover, mapping the broader CAV1 interactome, including adaptors, kinases, and trafficking regulators, could identify new therapeutic targets and biomarkers relevant to cancer progression and metastasis.

As most known mechanisms regulating CAV1 function are post-translational, exploring CAV1 at the genomic level remains crucial. *CAV1* Gene amplification was found in 13% of breast cancer cases with strong CAV1 expression [273]. Mutations in the *CAV1* gene have been identified in certain cancers. Emerging evidence suggests that some of these mutations can affect metastatic behaviour. Mutations in the CAV1 gene, particularly the P132L variant, have been identified in up to 16–19% of human breast cancers, especially in estrogen receptor α (ERα)-positive subtypes. The P132L mutant misfolds and accumulates in the Golgi apparatus, preventing proper caveolae formation and acting as a dominant-negative by trapping wild-type CAV1 intracellularly. This explains why many tumours harbour only a single mutated allele. P132L mutation disrupts normal tumour-suppressive activity of CAV1, while promoting cell migration, invasion, and metastasis. Gene expression profiling of P132L-expressing cells revealed upregulation of metastasis-related genes, including growth factors, extracellular matrix components, proteases, and signalling adapters. Additionally, CAV1-null mice exhibit mammary epithelial hyperplasia, suggesting that loss of CAV1 function contributes to early tumourigenesis [274–277]. Understanding the role of CAV1 mutations in cancer may lead to new therapeutic strategies. For example, targeting the signalling pathways activated by CAV1(P132L) could be a potential therapeutic approach. Indeed, incorporating a genomic perspective would enhance the depth of current CAV1 research.

Further investigating how mechanical stiffness regulates CAV1 is crucial for understanding tumour progression and metastasis. While mechanical stiffness is a well-documented inducer of CAV1 phosphorylation on tyrosine-14, this response is not entirely universal and appears to be context- and cell-type dependent. In many adherent cell types, increased substrate stiffness enhances focal adhesion maturation and cytoskeletal tension, which activates Src family kinases, leading to elevated pY14-CAV1. However, the magnitude and dynamics of this response can vary depending on cell lineage, differentiation state, and the presence of integrin or caveolae-associated signalling adaptors. Consequently, some types of cells can exhibit durotaxis and other negative durotaxis [43, 278]. Beyond Y14 phosphorylation, mechanical stiffness can also influence several other post-translational modifications and behaviours of CAV1, including ubiquitination (which may target CAV1 for lysosomal degradation in response to sustained mechanical stress or membrane tension) or palmitoylation, which affects membrane anchoring and trafficking, and may be regulated by mechanical inputs indirectly via oxidative or metabolic stress. Moreover, palmitoylation of CAV1 at Cys-156 controls its coupling to c-Src [279]. CAV1 ubiquitination at specific N-terminal lysine residues may play a key role in dynamically regulating its fate between degradation and secretion [280]. Furthermore, caveolae and membrane rafts provide a favourable platform for the recruitment of ubiquitin ligases that mediate the ubiquitination and subsequent degradation of CAV1 and TGFβ receptors. Specifically, CAV1 facilitates the internalisation of ubiquitinated TGFβ receptors at the plasma membrane and their transport to lysosomes, thereby attenuating TGFβ signalling. However, the deubiquitinase POH1 can reverse this process by reducing the ubiquitination of both CAV1 and TGFβ receptors, limiting their lysosomal degradation. In liver cancer cells, this stabilisation of TGFβ receptors enhances TGFβ signalling and promotes cell migration and invasion. These findings suggest that mechanical stretching of caveolae, which may reduce ubiquitination, could further influence this pathway, possibly affecting stromal TGFβ signalling and tumour-stroma interactions [210].

In conclusion, researching CAV1 holds great promise for uncovering mechanisms of metastasis, therapy resistance, and tumour-stroma communication. Understanding CAV1’s regulation, interactors, and signalling networks may lead to novel biomarkers and therapeutic approaches for aggressive and treatment-resistant cancers.

## Data Availability

No datasets were generated or analysed during the current study.
